# Diagnostic challenges and Gene-Xpert utility in detecting *Mycobacterium tuberculosis* among suspected cases of Pulmonary tuberculosis

**DOI:** 10.1371/journal.pone.0251858

**Published:** 2021-05-20

**Authors:** Shaila Kabir, M. Tanveer Hossain Parash, Nor Amalina Emran, A. B. M. Tofazzal Hossain, Sadia Choudhury Shimmi

**Affiliations:** 1 Faculty of Medicine and Health Sciences, Department of Medicine Based Disciplines, Universiti Malaysia Sabah, Sandakan, Malaysia; 2 Faculty of Medicine and Health Sciences, Department of Biomedical Science and Therapeutics, Universiti Malaysia Sabah, Sandakan, Malaysia; 3 Faculty of Medicine and Health Sciences, Borneo Medical and Health Research Centre, Universiti Malaysia Sabah, Sandakan, Malaysia; 4 Faculty of Medicine and Health Sciences, Department of Pathobiology and Medical Diagnostics, Universiti Malaysia Sabah, Sandakan, Malaysia; 5 Faculty of Medicine and Health Sciences, Tuberculosis Research Unit, Universiti Malaysia Sabah, Sandakan, Malaysia; 6 Faculty of Medicine and Health Sciences, Department of Surgical Based Disciplines, Universiti Malaysia Sabah, Sandakan, Malaysia; The University of Georgia, UNITED STATES

## Abstract

The incidence of pulmonary tuberculosis (PTB) can be reduced by preventing transmission with rapid and precise case detection and early treatment. The Gene-Xpert MTB/RIF assay is a useful tool for detecting *Mycobacterium tuberculosis* (MTB) with rifampicin resistance within approximately two hours by using a nucleic acid amplification technique. This study was designed to reduce the underdiagnosis of smear-negative pulmonary TB and to assess the clinical and radiological characteristics of PTB patients. This cross-sectional study included 235 participants who went to the Luyang primary health care clinic from September 2016 to June 2017. The demographic data were analyzed to investigate the association of patient gender, age group, and ethnicity by chi-square test. To assess the efficacy of the diagnostic test, the sensitivity, specificity, positive predictive value (PPV), negative predictive value (NPV), and accuracy were calculated. The area under the curve for sputum for both AFB and gene-Xpert was analyzed to compare their accuracy in diagnosing TB. In this study, TB was more common in males than in females. The majority (50.71%) of the cases belonged to the 25–44-year-old age group and the Bajau ethnicity (57.74%). Out of 50 pulmonary TB cases (smear-positive with AFB staining), 49 samples were positive according to the Gene-Xpert MTB/RIF assay and was confirmed by MTB culture. However, out of 185 smear-negative presumptive cases, 21 cases were positive by Gene-Xpert MTB/RIF assay in that a sample showed drug resistance, and these results were confirmed by MTB culture, showing resistance to isoniazid. In comparison to sputum for AFB, Gene-Xpert showed more sensitivity and specificity with almost complete accuracy. The additional 21 PTB cases detection from the presumptive cases by GeneXpert had significant impact compared to initial observation by the routine tests which overcame the diagnostic challenges and ambiguities.

## Introduction

Tuberculosis (TB) is caused by the single infectious agent *Mycobacterium tuberculosis*, and it is one of the oldest known chronic infectious diseases. It is among the top ten leading causes of death worldwide [1]. TB usually affects the lungs, but it can also affect other parts of the body, such as the brain, spine, and many other organ systems. The most common form is pulmonary TB, which is easily spread by aerosol droplets. If another person inhales air containing these droplet nuclei, the probability of getting infected is very high. The chance of transmissibility increases if there is a delay in disease detection and treatment initiation [2,3].

According to the World Health Organization (WHO) global tuberculosis report, there were approximately 10.0 million new TB cases worldwide in 2019, but only 7.0 million cases were notified, for a large gap in TB notifications. There were approximately half a million new cases of rifampicin-resistant TB (of which 78% had multidrug resistance) globally, but only 206 030 new cases of MDR/RR-TB were detected and notified in 2019. There were an estimated 1.2 million TB deaths, with an additional 208 000 deaths resulting from TB coinfection with HIV [1].

The incidence of tuberculosis cases rose from 11,702 in 1990 to approximately 27,000 in 2015 in Malaysia [4]. In that country, 25 173 TB cases were reported, with an incidence rate of 92 per 100 000 population in 2018. Although 10% of the Malaysian population resides in Sabah state, 19.7% (33 193 TB cases) of the total TB cases in Malaysia were notified from 2012–2018, for a notification rate of 128 cases (range: 120–133) per 100 000 people in Sabah. The highest number, 904 TB cases among 5008 TB cases, was notified in Kota Kinabalu, the Sabah state capital, in 2018 [5].

The provision of adequate tuberculosis control is incomplete despite quality-assured services due to delayed case detection, leading to suffering, death, and disease transmission [6,7]. The conventional methods for TB detection are smear microscopy, culture, sensitivity testing, and chest radiography. TB culture and sensitivity testing are the gold standards for diagnosis [8–10]. The use of sputum smear microscopy for detecting tuberculosis is a cheap diagnostic tool but has lower sensitivity (20–78.3%) [11]. Compared to sputum smear-positive TB, sputum smear-negative TB is less infectious [12,13], and patients with smear-negative but culture-positive pulmonary TB are capable of transmitting *M*. *tuberculosis* [11,13–16]. A study conducted by Tostmann et al. (2008) suggested that patients with smear-negative pulmonary TB cause 12.6% of TB transmission in the Netherlands. Therefore, the researchers recommended that, in addition to assisting patients with smear-positive TB, contact investigations should include smear-negative TB in countries with a low TB burden and sufficient public health resources [17].

For the rapid detection of MDR-TB, the WHO has endorsed the Gene-Xpert MTB/RIF assay, which can detect *Mycobacterium tuberculosis* (MTB) and resistance to rifampicin within approximately two hours by using a nucleic acid amplification technique [18]. It is necessary to use better diagnostic tools for TB detection and drug resistance testing with proven efficacy and affordability [18–20]. Thus, a rapid and accurate diagnostic tool is required for the rapid diagnosis of both smear-positive and smear-negative TB to ensure early treatment, which would reduce the suffering from TB and cease its spread. According to the World Health Organization (WHO) global tuberculosis report, as of 2020, there is a large gap (2.9 million) in TB notifications. One of the major causes of this gap is the lower efficacy of the diagnostic algorithm [1]. According to Malaysian clinical practice guidelines, the initial diagnostic tool for PTB is smear microscopy with Ziehl-Neelsen staining (AFB staining), which has less sensitivity. It is hypothesized that a given percentage of TB cases at this TB center in the Luyang Primary health care clinic are underdiagnosed, and that they could be detected better by using the Gene-Xpert system. This statement is suggestive of the improved detection and notification rate of MDR/RR-TB from 156,205 in 2018 to 206,030 in 2019 [1]. This improvement is most likely due to using better diagnostic methods [21]. This study was designed to reduce the underdiagnosis of smear-negative pulmonary TB and to assess the clinical and radiological characteristics of PTB patients.

## Materials and methods

This cross-sectional study was conducted from September 2016 to June 2017 at the TB outpatient department of Klinik Kesihatan Luyang, Kota Kinabalu, Sabah, Malaysia. This facility is a primary health care clinic for the local communities, and the TB center is a referral center in Kota Kinabalu, Sabah.

### Selection criteria

The patients who fulfilled the following criteria were included in the study:

- Presumptive TB cases at the first visit (within two weeks of presentation at the health facilities)
- Returnees for retreatment
- History of contact with TB patients

Exclusion Criteria:

- Children under 15 years of age
- Individuals who had started taking anti-TB drugs
- Individuals who were diagnosed with HIV (there is a dedicated center in Malaysia to treat patients with HIV)

### Sample size

According to Flahault et al. [22], the required numbers of cases and controls are 178 and 50, respectively, an expected level of sensitivity of 95% and the minimum acceptable lower confidence limit is 87% with 95% probability, where the following formula was used to calculate the number [22]:

$$N_{controls}=N_{cases}\left[\left(1‐Prev\right)/Prev\right]$$

Here, *N* = number, Prev = prevalence, (prevalence in Sabah for TB = 0.22) [23],

Case: Participants who were presumed to have TB because of clinical or radiographic suspicion of tuberculosis but were found to be smear-negative. Control: Participants who were newly diagnosed with smear-positive pulmonary TB as detected by the clinic.

A total of 280 subjects were initially selected by stratified random sampling, but 45 patients were not accepted for final statistical analysis due to incomplete information or incomplete diagnostic investigations. All of those patients were smear-negative and Gene-Xpert negative but missing a few other analyses, such as chest radiographs or other laboratory investigations. Out of these 235 patients, 50 were sputum-positive for AFB, and 185 were presumptive cases of TB but sputum-negative for AFB. All the patients were enrolled in the study before the attending physician received any routine laboratory tests.

### Patient enrollment

The study patients were enrolled at the TB outpatient department of Klinik Kesihatan Luyang, a primary health care clinic and primary care provider to the local communities, and the TB center is a referral center in Kota Kinabalu, Sabah, Malaysia.

Patients with suspected pulmonary tuberculosis based on the presence of risk factors, symptoms and signs at this clinic were eligible for enrollment in this cross-sectional study, which was conducted from September 2016 to June 2017. Patient enrollment was performed during normal clinical hours on weekdays, following simple random sampling. Patients with presumptive pulmonary tuberculosis were enrolled during regular clinical hours on weekdays. Patients suffering from a cough of 2–3 weeks in duration, who were coughing up blood and experiencing a prolonged fever, loss of appetite, loss of weight, and night sweating, were considered presumptive, to rule out pulmonary tuberculosis [23].

## Informed consent

Written informed consent was obtained from potentially eligible participants after verbal and written explanations were provided with information about the study by the research staff.

## Human research ethics

The study was approved by the Faculty of Medicine and Health Sciences, UMS, Ethics Committee [JKEtika 3/16(2)] and the Malaysian Medical Research & Ethics Committee National Medical Research Register (NMRR number: 16-810-30378).

### Detailed data collection steps

*Clinical*, *radiological*, *and routine laboratory investigations*. For each patient, the detailed history of symptoms, signs, and risk factors (HIV patient, previous history, family members, or close friends with TB, suffering from renal failure, rheumatoid arthritis, diabetes mellitus, COPD, smoker, and low BMI ≤ 18.5) was documented.

According to the clinical protocol, three samples of sputum were taken for AFB staining on three consecutive days from each patient. A chest X-ray was taken for radiological evaluation, and blood samples were drawn for routine hematological and biochemical assays, which were analyzed at the clinic’s laboratory. On the second or third day, one sputum sample was taken for the Gene-Xpert MTB/RIF assay. *Mycobacterium tuberculosis* culture and drug sensitivity tests from the sputum samples were performed for the AFB- or Gene-Xpert MTB/RIF-positive tuberculosis cases.

*Sputum sample collection*. Sputum samples were collected for smear microscopy spot sampling, and the second samples were collected on the second day in the early morning for smear microscopy and Gene-Xpert assay. Sputum was obtained after a deep, productive cough (a satisfactory quality implies the presence of mucoid or mucopurulent material). The mouth was rinsed with water before the specimen was produced. For optimal results, the subjects needed to cough out sputum into clean and sterile specimen containers that had been labeled accordingly. Sputum was never collected in confined areas, such as toilets or laboratories. It was performed in a separate open-air designated area (which rapidly dilutes aerosols, and UV light rapidly inactivates the bacilli), away from other people. As with the protocol, no one could stand in front of the patient during expectoration. [9,24]

*Sputum sample storage and transport*. After the subjects had coughed out the sputum, the specimen containers were screw-capped tightly to avoid spillage. The specimens were kept cool (4–15°C) at all times until processing. All the specimens were delivered for Gene-Xpert analysis within 8 hours [24]

*Smear Preparation and Ziehl-Neelsen staining*. By using a transfer pipette, ~100 μl (2 drops) of sputum specimen was placed onto the slide and spread. For at least 2 hours, the smear was air-dried at a temperature between 65°C and 75°C. After the slide the flooded with carbol fuchsin, it was heated to steaming for 5 minutes. After the stain was washed off with distilled water, the slide was flooded with 3% acid-alcohol for 2–3 minutes. The acid-alcohol was washed off with distilled water, and the slide was tilted to drain. After that, the slide was again flooded with methylene blue, allowed to stand for 1–2 minutes, and washed of methylene blue with distilled water. The slide was then drained and air-dried. Using a bright field microscope, the Ziehl-Neelsen smears were examined with a 100X oil objective (10X eyepiece for a total of 1000X magnification). Ziehl-Neelsen stains acid-fast organisms red, and the background debris stains blue [24].

*MTB culture in Lowenstein-Jensen (LJ) media*. LJ media was used to isolate and grow MTB. The sputum samples were inoculated and decontaminated by using N-acetyl-L-cysteine (NALC) with Na citrate solution. NaOH was used to concentrate the sputum specimens. After that, each prepared sample was inoculated for approximately one week in a *Mycobacteria* Growth Indicator Tube (MGIT). A buff-colored, dry colony of MTB growth was obtained and stored in a dark and cold place. The growth was recorded weekly. If there was no growth from weeks 1 to 8, the samples were recorded as showing no growth. If there was growth at any reading interval, reincubation was performed, and if the count increased significantly, the weekly culture continued to be read until the growth stabilized. Then, all the first-line drugs (rifampicin, isoniazid, ethambutol, pyrazinamide, and streptomycin) were tested in the MGIT 960 system. Additionally, fluoroquinolones (levofloxacin, gatifloxacin, ofloxacin, and moxifloxacin) and injectable drugs (amikacin, kanamycin, and capreomycin) were tested in the MGIT 960 system [24].

*Sputum samples for the Gene-Xpert MTB/RIF assay*. For this study purpose, sputum samples were obtained in sputum cups and kept in a cold box inside a biosafety bag. On the same day, the resulting samples were sent to the Tuberculosis Laboratory of the University Malaysia Sabah. The sputum samples were processed according to the manufacturer’s protocol for the Gene-Xpert MTB/RIF assay. The sample reagent was added to unprocessed sputum at a 2:1 ratio in a 15 ml falcon tube, and the tube was manually agitated twice during a 15-minute incubation period at room temperature. Then, 2 ml of the inactivated material was transferred to the test cartridge with a sterile disposable pipette (provided with the kits). The cartridges were loaded into the Gene-Xpert machine. The entire laboratory procedure was performed using biosafety measures according to the standard laboratory protocol. The interpretation of data from the MTB/RIF tests was software-based and not user-dependent [20].

*Pulmonary tuberculosis diagnostics and case definitions*. In this study, the standard used for the TB diagnosis was a composite derived from clinical, radiological, and bacteriological or molecular evidence. All the patients were confirmed to have at least one positive, either bacteriological or molecular evidence: sputum AFB, MTB culture, or Gene-Xpert assay.

*Statistical analysis*. The demographic data were analyzed to investigate the association of patient gender, age group, and ethnicity by chi-square test using SPSS version 22.0. To assess the efficacy of the diagnostic test, the sensitivity, specificity, positive predictive value (PPV), negative predictive value (NPV), and accuracy were calculated using the following formulae in Microsoft Excel:

Sensitivity = a/(a+c),

Specificity = d/(b+d)

PPV = a/(a+b)

NPV = d/(c+d)

Accuracy = (a+b)/(a+b+c+d)

where a = true positive, b = false positive, c = false negative and d = true negative.

The area under the curve for sputum for both AFB and gene-Xpert was analyzed using the same SPSS version to compare their accuracy in diagnosing TB.

## Results

In this cross-sectional study, more than half were male participants, and their ages ranged from 15 to 65 years. From an ethnic point of view, most participants belonged to the Bajau ethnicity (Table 1).

**Table 1:** Distribution of participants by gender, age group, and ethnicity with regards to TB (n = 235).

		Non-TB	TB	Level of significance (p*)
**Gender**	Male	70 (42.68%)	50 (70.42%)	<0.01
	Female	94 (57.32%)	21(29.58%)	
**Age**	15–24 years	9 (5.49%)	11 (15.49%)	<0.05
	25–44 years	75 (45.73%)	36 (50.71%)	
	45–64 years	52 (31.71%)	17 (23.94%)	
	≥65 years	28 (17.07%)	7 (9.86%)	
**Ethnicity**	Bajau	53 (32.32%)	41(57.74%)	<0.01
	Chinese	36 (21.95%)	8(11.27%)	
	KadazanDusun	31 (18.90%)	7 (9.86%)	
	Others**	44 (26.83%)	15(21.13%)	
	**Total**	164 (100%)	71 (100%)	

* Probability from Chi-square analysis. P<0.05 was considered to be significant.

** Malay, Rungus, Bugis, Suluk, Sino, Indian, Brunei, Sungai, Timur, Ubian, Tidung, and Mixed ethnicity.

**Fig 1:**
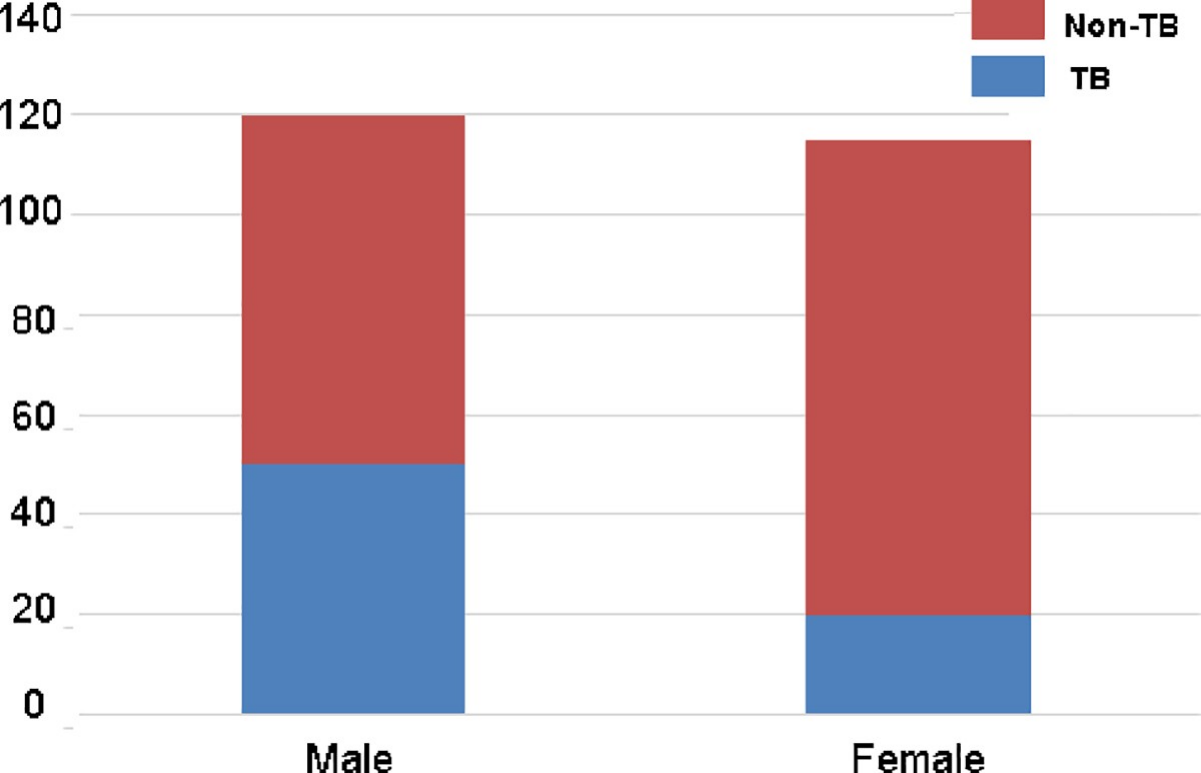
Distribution of tuberculosis by sex (n = 235).

**Fig 2:**
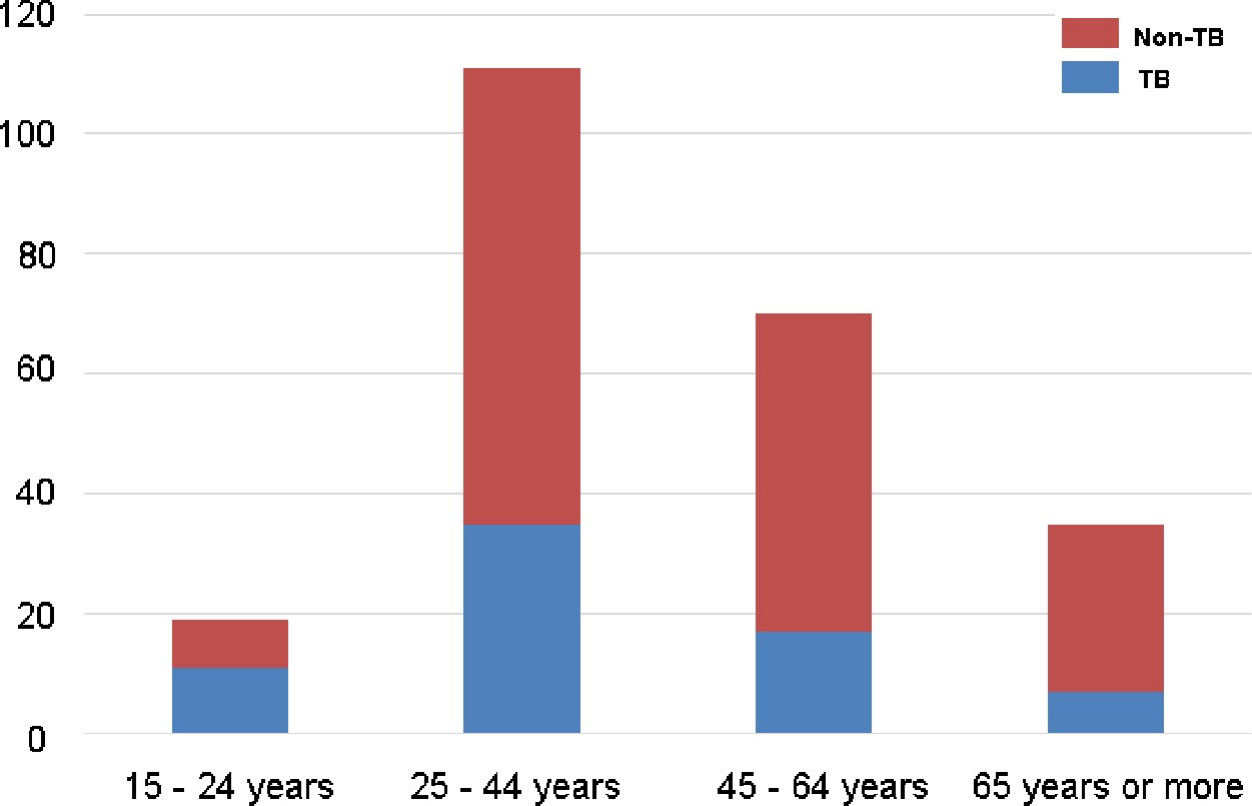
Distribution of tuberculosis with respect to age (n = 235).

**Fig 3:**
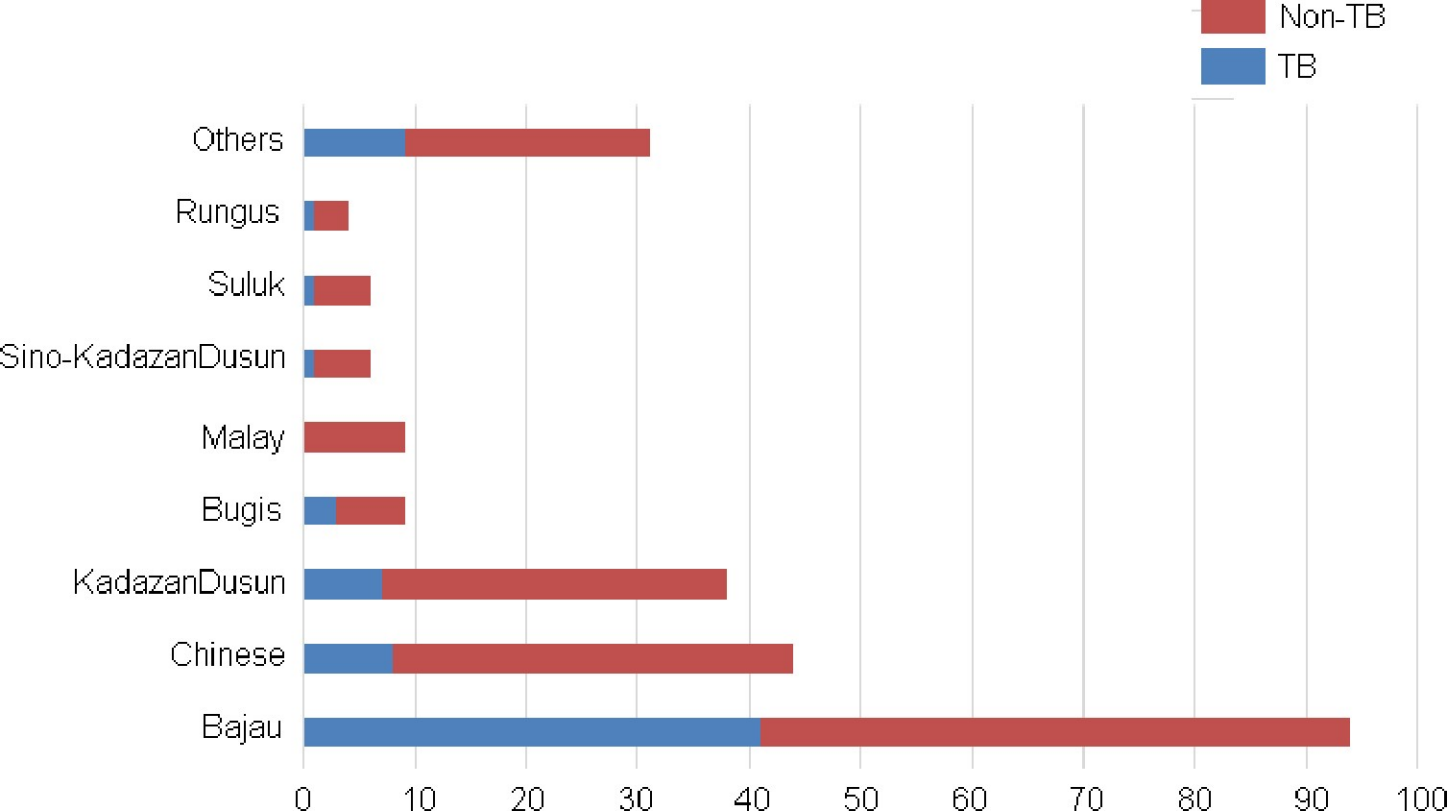
Distribution of TB with respect to ethnicity (n = 235).

From Fig 1, it is evident that TB was more common in males than in females. The majority (50.71%) of the cases belonged to the 25–44 year-old age group (Fig 2) and the Bajau ethnicity (Fig 3).

Although all the participants had symptoms, persistent cough, loss of appetite and loss of body weight showed significant differences between the presumptive TB cases and smear-positive controls (Table 2). Having a low BMI and a history of close contact with TB patients were significant risk factors (Table 3). Almost all smear-positive controls had shadows in their chest X-rays (Table 4).

**Table 2:** Distribution of symptoms among case and control group (n = 235).

Symptoms		Case (Presumptive cases)	Control (Smear Positive)	Level of significance (p*)
Persistent cough	Present	184	47	<0.05^a^
	Absent	1	3	
Coughing blood	Present	38	14	>0.05
	Absent	147	36	
Prolonged fever	Present	64	21	>0.05
	Absent	121	29	
Loss of appetite	Present	60	23	<0.05
	Absent	125	27	
Loss of weight	Present	59	25	<0.05
	Absent	126	25	
Night Sweating	Present	25	8	>0.05
	Absent	160	42	

* Probability from Chi-square analysis. P<0.05 was considered to be significant.

^a^ Probability from Fisher’s exact test as more than one cells had expected values less than 5.

**Table 3:** Distribution of risk factors among case and control group (n = 235).

Risk factors		Case (Presumptive cases)	Control (Smear Positive)	Level of significance (p*)
Previous history of TB	Present	20	4	>0.05^a^
	Absent	165	46	
History of close contact with TB cases	Present	77	24	<0.05
	Absent	108	26	
Smoking	Present	47	14	<0.05
	Absent	138	36	
Diabetes mellitus	Present	23	5	>0.05^a^
	Absent	162	45	
Chronic obstructive pulmonary disease	Present	4	0	>0.05^a^
	Absent	181	50	
End stage renal failure	Present	1	0	>0.05^a^
	Absent	184	50	
BMI <18.5	Present	99	35	<0.001
	Absent	86	15	

* Probability from Chi-square analysis. P<0.05 was considered to be significant.

^a^ Probability from Fisher’s exact test as more than one cells had expected values less than 5.

**Table 4:** Distribution of radiological evidence among case and control group.

Chest X-ray	Case (Presumptive cases)	Control (Smear Positive)	Level of significance (p*)
**No lesion**	157	1	<0.001
**With lesions**	28	49	
**Total**	185	50	

* Probability from Chi-square analysis. P<0.05 was considered to be significant.

49 samples were positive according to the Gene-Xpert MTB/RIF assay and was confirmed by MTB culture. Notably, out of 185 presumptive cases of TB (smear-negative with AFB staining but clinical and/or radiographic suspicion of tuberculosis), 21 cases were positive by gene-Xpert MTB/RIF assay in which a sample showed drug resistance, and the result was confirmed by MTB culture, showing resistance to isoniazid (Tables 5 and 6). The additional 21 PTB cases detection from the presumptive cases by GeneXpert had significant impact compared to initial observation by the routine tests which overcame the diagnostic challenges and ambiguities.

**Table 5:** Gene-Xpert results compared to sputum for AFB (n = 235).

Diagnostic test		Tuberculosis confirmed by MTB Culture		Total
		Present	Absent	
Sputum for AFB	Positive	49 (a)	1(b)	50
	Negative	22 (c)	163 (d)	185
Gene-Xpert	Positive	70^†^(a)	1(b)	71
	Negative	1 (c)	163(d)	164
Total		71	164	235

*a = true positive, b = false positive, c = false negative, d = true negative^†^; one person was diagnosed with MDR-TB.

**Table 6:** Drug resistance and sensitivity (n = 71).

	MDR-TB	Drug sensitivity
Gene-Xpert@MTB/RIF	1*	
MTB culture	1**	70

* The same patient was confirmed to be resistant to anti-TB drugs by MTB culture.

**Resistance to isoniazid according to drug sensitivity testing.

In this study, Gene-Xpert showed 98.59% sensitivity, 99.39% specificity, 98.59% positive predictive value, and 99.39% negative predictive value with an accuracy of 99.15% compared to the sputum AFB, which displayed values of 69.01%, 99.39%, 98%, 88.11% and 90.21%, respectively (Table 7).

**Table 7:** Sensitivity, specificity, predictive values, and accuracy of sputum tested by AFB and Gene-Xpert (n = 235).

Diagnostic test		Proportion	N	SE	Width	95% CI	
						Upper	Lower
**Sputum for AFB**	**Sensitivity**	0.6901	71	0.0548	0.1075	0.5826	0.7977
	**Specificity**	0.9939	164	0.0061	0.0119	0.9820	1.0058
	**PPV**	0.98	50	0.0198	0.0388	0.9412	1.0188
	**NPV**	0.8811	185	0.0238	0.0466	0.8344	0.9277
	**Accuracy**	0.9021	235	0.0194	0.0380	0.8641	0.9401
**Gene-Xpert**	**Sensitivity**	0.9859	71	0.014	0.0274	0.9585	1.0133
	**Specificity**	0.9939	164	0.0061	0.0119	0.9820	1.0058
	**PPV**	0.9859	71	0.014	0.0274	0.9585	1.0133
	**NPV**	0.9939	164	0.0061	0.0119	0.9820	1.0058
	**Accuracy**	0.9915	235	0.006	0.0117	0.9797	1.0032

## Discussion

In this study, among 120 male and 115 female participants, the males were affected significantly more than the females (p<0.01). This sex disparity among the reported PTB case findings corresponds to the findings by Goroh MMD et al [5], Behr MA et al [14], Hernández-Garduño E et al [15], Tostmann A et al [17], Linguissi LS et al [25], Zhang X et al [26] and Smiljić S et al [27].

Although all the age groups were affected, PTB was comparatively more common (p<0.05) in the 25–44 year-old group. Goroh MMD et al [5], Zhang X et al [26] and Smiljić S et al [27] found the same trend in their studies, in which the same age group had the highest number of TB patients, and Linguissi LS et al [25] reported that the median (IQR) age for PTB patients was 32 (25–46) years. By contrast, Tostmann A et al [17] reported the highest affected age group to be 15–34 years old, which is partly within the 25–44 year age group but comparatively lower. Behr MA et al [14] and Hernández-Garduño E et al [15] found a mean age of approximately 50 years.

This study also demonstrated the highest incidence of PTB among the Bajau ethnic groups, followed by the Chinese, KadazanDusun, and other ethnic groups. Although this study showed a very significant (p<0.01) difference among different ethnicities, the findings could not be compared to those of Goroh MMD et al [5], because they did not publish their results according to ethnicity. However, in a study done in the Kudat Division, Sabah, Roslie R et al [28] found that thirty-three percent of the participants had more than 6 household members and on average household income was below RM1000. The Bajau ethnic groups were among the major ethnic groups in this research. According to Clark M et al [29] and Narasimhan P et al [30], socioeconomic conditions such as overcrowding, and poverty are the known contributors for PTB.

Among the 71 diagnosed cases of PTB, all were symptomatic, with the majority displaying risk factors and clinical signs. Persistent cough, coughing up blood, prolonged fever, loss of appetite,

loss of weight, and night sweating were the chief complaints among the study participants. There was a significant difference (p<0.05) between the smear-positive controls and presumptive cases in presenting persistent cough, loss of appetite, and loss of weight. Smiljić S et al [27] and Appleton SC [31] found cough, fever, weight loss, and night sweats to be the prevalent symptoms.

Although the previous history of TB, history of close contact with TB cases, smoking, diabetes mellitus, chronic obstructive pulmonary disease, end-stage renal failure, and BMI <18.5 were considered to be the risk factors for contracting PTB, only history of close contact, smoking, and BMI<18.5 showed significant differences between the case and control groups. This finding corresponds to Narasimhan P et al [30], Zhang CY et al [32], Lin HH et al [33], and Zhang H et al [34]. In this study, none of the participants had HIV as a risk factor, reflecting the finding of William T et al. [2], in which the rate of HIV-TB coinfection was only 1.7%. According to the HIV treatment guidelines, any patient with HIV is treated in a dedicated HIV center. Once a patient is smear-positive for TB, that individual is also tested for HIV. If the test result is positive for HIV, the patient is referred to the HIV-dedicated center, where the patient receives TB treatment. This might be the reason for the lack of HIV-TB coinfection cases in this study because it was performed in a primary TB center.

The chest X-rays (CXR) of the participants revealed minimal and moderate lesions in 77 persons, in which 69 people were diagnosed with PTB. Only two TB cases did not show any change in the CXR. The studies conducted by Smiljić S et al [27] and Appleton SC [31] also had similar findings. The WHO [35], in its "End TB Strategy," mentioned that CXR has high sensitivity but poor specificity. However, if it is used together with Gene-Xpert, it can be a handy triage tool. The WHO further proposed using the triage algorithm (Algorithm 7 and Algorithm 9), where it showed that having a cough or any TB symptoms followed by CXR followed by Xpert MTB/RIF testing is much more cost-effective, yielding more true positive results and fewer false-positive results compared to having a cough or any TB symptoms followed by CXR followed by microscopy [35].

Though few symptoms, risk factors, and radiological evidence were significantly different among the smear-positive controls and presumptive TB cases, there were plenty of presumptive TB cases having similar findings. These findings created diagnostic challenges created the possibility of missing possible TB detection. To overcome this challenge gene-Xpert MTB/RIF assay was conducted. Out of 185 presumptive smear-negative TB cases, 21 cases were positive by gene-Xpert MTB/RIF assay. Without gene-Xpert assay, we could have missed thirty percent of PTB cases.

**Fig 4:**
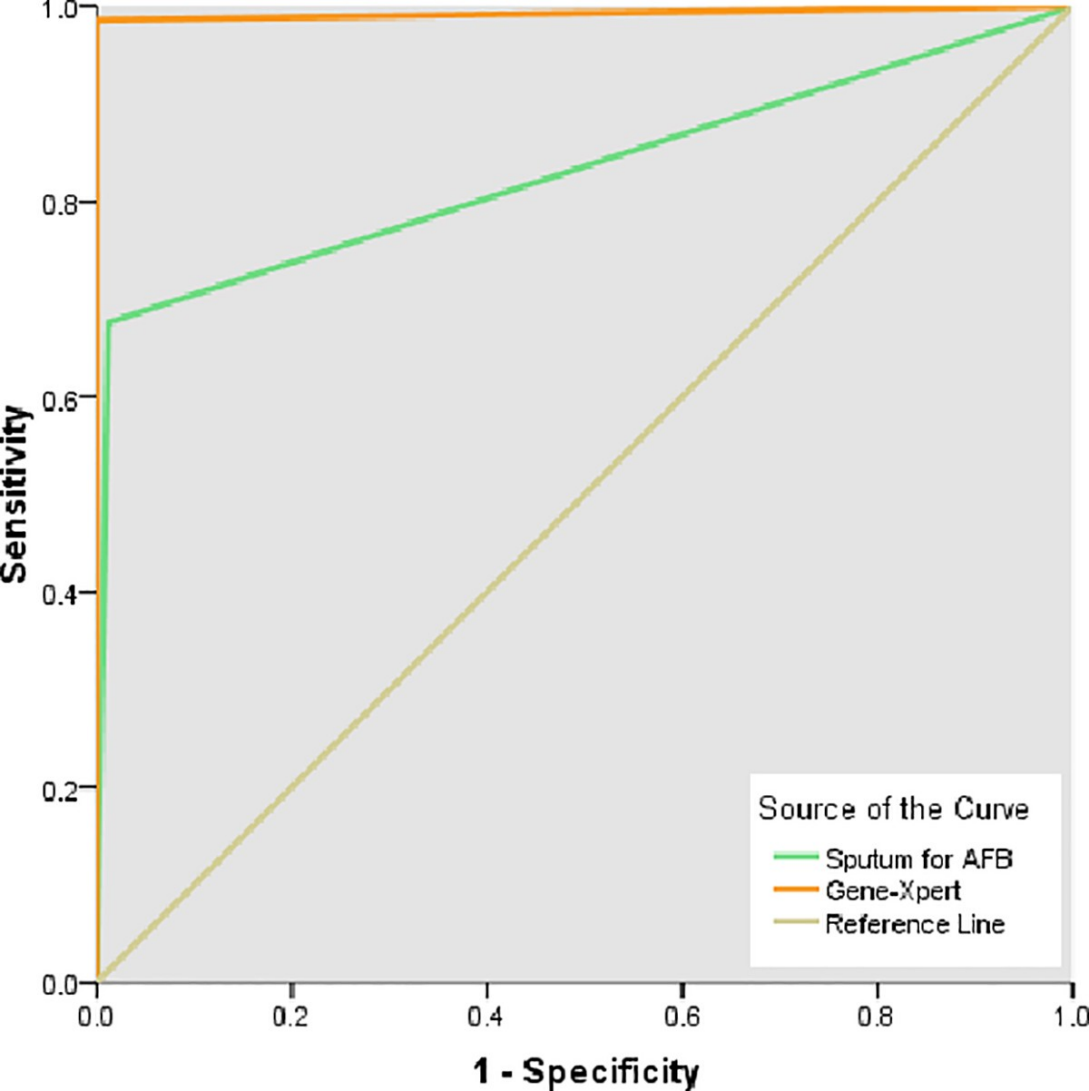
ROC curves for Sputum for AFB and Gene-Xpert.

This study compared the performance of two diagnostic approaches: sputum AFB staining and Gene-Xpert MTB/RIF, in which an MTB culture was set as the gold standard. In comparison to sputum for AFB, Gene-Xpert showed more sensitivity and specificity with almost complete accuracy in this study (Table 8 and Fig 4). Similar findings were observed by Zeka AN et al [36], Iram S et al [37], Prakash AK et al [38], and Dunn JJ et al [39]. Due to cost-effectiveness concerns, sputum smear microscopy with Ziehl-Neelsen (ZN) staining is widely used. MTB detection using this method has high specificity, and it does not require sophisticated equipment [40,41]. Smear microscopy results can be obtained within 2 h; however, smear microscopy is less sensitive because it requires 5000–10,000 bacilli per mL of sputum to show a positive result. Almost 13% of TB transmission occurs in smear-negative, culture-positive TB patients. Therefore, healthy individuals are at risk of MTB infection leading to active TB development when coming in close contact with sputum-negative TB suspects. Moreover, this test requires a 3-day early morning sputum specimen collection protocol to enhance its sensitivity. In addition to the lower sensitivity of sputum smear microscopy, it cannot differentiate MTB from the MTB complex [41,42]

**Table 8:** Area under the curve of sputum for AFB and Gene-Xpert (n = 235).

Diagnostic tests	Area	Std. Error^a^	Asymptotic Sig.^b^	Asymptotic 95% Confidence Interval	
				Lower Bound	Upper Bound
Sputum for AFB	.832	.035	.000	.763	.900
Gene-Xpert	.993	.008	.000	.977	1.009

a. Under the nonparametric assumption.

b. Null hypothesis: true area = 0.5.

A culture technique using Lowenstein Jensen (LJ) medium for mycobacterial growth is considered as the gold standard method for TB detection, and it takes a longer amount of time, which is usually 3–4 weeks with high sensitivity, but it requires a biosafety level III laboratory. The efficiency of MTB culture using LJ medium has been demonstrated to detect MTB when 10 viable bacilli per mL of sputum were present [43]. *M*. *tuberculosis* culture for detecting resistant strains generally takes 3–8 weeks. Drug sensitivity testing usually takes approximately 2–4 weeks in solid media and 1 week in broth media [44,45].

In comparison, the Gene-Xpert MTB/RIF assay can detect *M*. *tuberculosis* and rifampicin-resistant strains simultaneously and directly from clinical specimens within 2 hours [36,46,47]. Two multicenter studies have shown that *M*. *tuberculosis* was detected in all smear-positive and in three-quarters of smear-negative cases by using a single Gene-Xpert assay [48,49]. These authors reported Gene-Xpert sensitivities of 57%-76.9% and 98%-100% in smear-negative, culture-positive and smear-positive, culture-positive respiratory specimens, respectively, with a similar specificity of 99%-100% [36,48–52].

The top eight TB endemic countries are India, Indonesia, China, the Philippines, Pakistan, Nigeria, Bangladesh, and South Africa. All the research showed similar results compared to our research outcomes. These studies concluded that the application of the Gene-Xpert MTB/RIF assay showed dramatic improvements for the rapid detection of tuberculosis and drug resistance, which offers promising advancements in TB control programs [53–60].

The major limitation of this study is that not all the patients (both smear- and Gene Xpert negative) were assessed by MTB culture due to financial constraints. Furthermore, this was a single-center study.

## Conclusions

The study results highlight the utility of Gene-Xpert tests for an early specific diagnosis, which is far better than using conventional AFB smears as a first diagnostic tool. Patients with significant demographic profile and clinical characteristics, should undergo Gene-Xpert assay to overcome diagnostic dilemma. In addition, because this Gene-Xpert MTB/RIF assay can quickly identify possible multidrug-resistant TB (MDR TB), it allows TB patients to start effective treatment much sooner than if they had to wait for results from other types of drug susceptibility testing. Notably, the patients who did not have TB disease as identified by this assay could avoid unnecessary treatment and isolation in hospitals.

## Supporting information

S1 Data (XLSX)
